# UV light and the ocular lens: a review of exposure models and resulting biomolecular changes

**DOI:** 10.3389/fopht.2024.1414483

**Published:** 2024-09-05

**Authors:** Emily R. MacFarlane, Paul J. Donaldson, Angus C. Grey

**Affiliations:** Department of Physiology, School of Medical Sciences, New Zealand National Eye Centre, University of Auckland, Auckland, New Zealand

**Keywords:** lens, UV light, cataract, UV-A, UV-B, oxidative stress, UV filter

## Abstract

UV light is known to cause damage to biomolecules in living tissue. Tissues of the eye that play highly specialised roles in forming our sense of sight are uniquely exposed to light of all wavelengths. While these tissues have evolved protective mechanisms to resist damage from UV wavelengths, prolonged exposure is thought to lead to pathological changes. In the lens, UV light exposure is a risk factor for the development of cataract, which is a condition that is characterised by opacity that impairs its function as a focusing element in the eye. Cataract can affect spatially distinct regions of the lens. Age-related nuclear cataract is the most prevalent form of cataract and is strongly associated with oxidative stress and a decrease in the antioxidant capacity of the central lens region. Since UV light can generate reactive oxygen species to induce oxidative stress, its effects on lens structure, transparency, and biochemistry have been extensively investigated in animal models in order to better understand human cataract aetiology. A review of the different light exposure models and the advances in mechanistic understanding gained from these models is presented.

## 1 Introduction

### 1.1 The cataract epidemic

Our sense of sight is critically dependent on the ability of the ocular lens to maintain its transparent and refractive properties over many decades of life. Failure to maintain lens transparency results in opacification of the lens due to the scattering of transmitted light rays. Lens opacification, or cataracts, are the leading cause of vision impairment and blindness worldwide (1), accounting for around half of all forms of vision loss (2). While cataract is a multi-factorial pathology, with genetics, increasing age, diabetes, and environmental factors such as exposure to cigarette smoking (3) and alcohol use (4) all contributing to its development, exposure to sunlight (UV radiation) is also a major risk factor (5–7), which can exacerbate different types of cataract.

**Figure 1:**
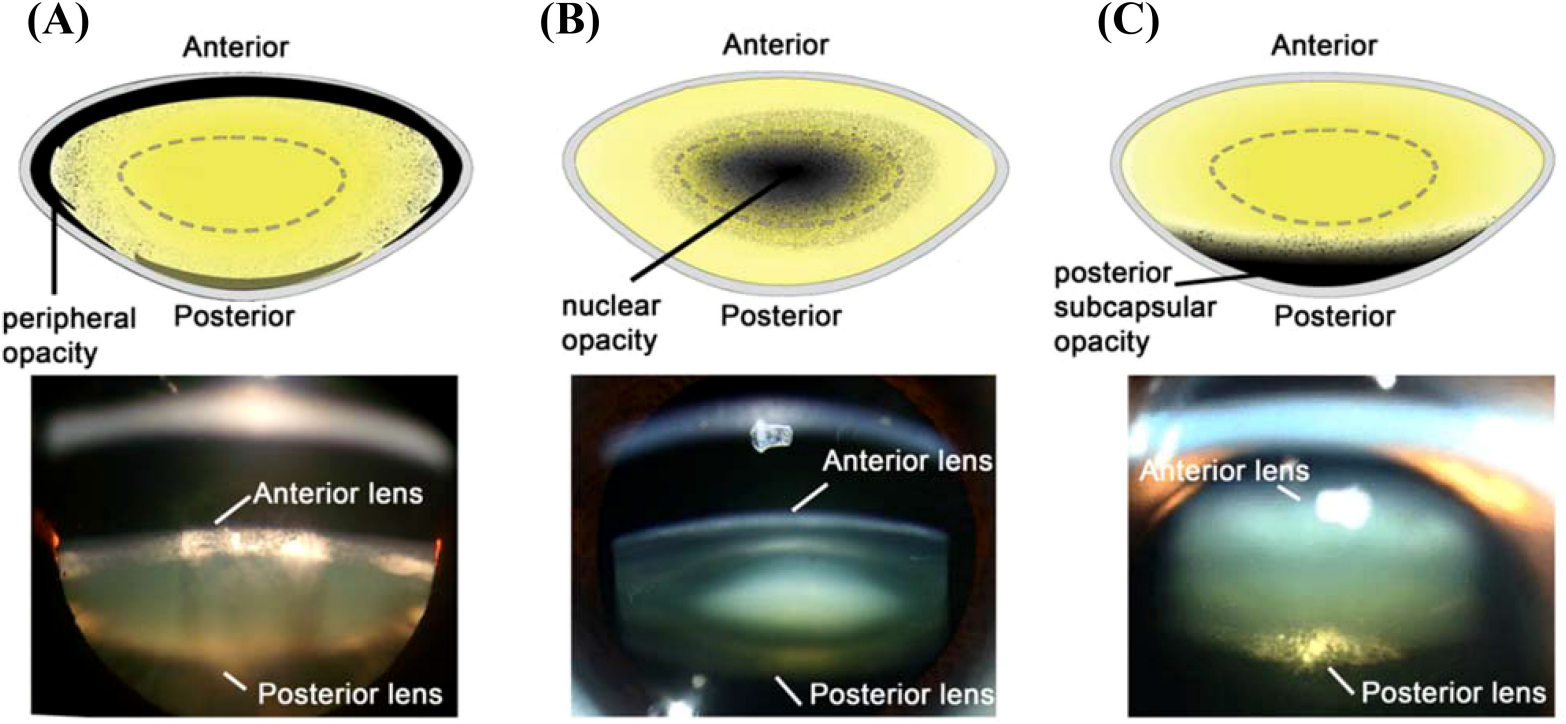
Schematic diagrams (Top) and Scheimpflug slit-lamp photographic (bottom) images of the three main types of cataracts. **(A)** cortical cataract, from Uspal NG, Schapiro ES (2011). Cataracts as the initial manifestation of type 1 diabetes mellitus. Paediatric Emergency Care. 27 (2): 132–4. **(B)** Nuclear cataract, from Ophthalmic Atlas Images by EyeRounds.org. **(C)** Posterior subcapsular cataract, from Chaudhary M, Shah DN, Chaudhary, RP (2017). Scleritis and Takayasu’s disease. Nepalese Journal of Ophthalmology (18): 170–174. Reproduced with permission from MDPI under Creative Commons Attribution (CC BY 4.0).

Cortical cataract, the second most prevalent form of cataract, occurs earlier than age-related nuclear (ARN) cataract (8–10), and progresses slowly before manifesting as tissue damage in the outer cortex of the lens (**Figure 1A**) (11). In contrast, ARN cataract in the human lens (**Figure 1B**) occurs when the intrinsic repair and protection mechanisms that exist to mitigate the effects of oxidative stress slowly deteriorate or become ineffective (12). Under oxidative stress conditions, thiol groups of proteins are easily oxidised to form protein mixed disulfides with oxidised glutathione (PSSG), cysteine (PSSC), and eventually, protein:protein cross-links (PSSP) (12). This accumulated damage can change protein structure and function, and leads to protein aggregation and insolubilisation (13, 14), which causes the light scattering that is characteristic of ARN cataract. Posterior subcapsular cataracts (**Figure 1C**) are characterised by dysplasia of the equatorial epithelial cells (15). On their own, they are relatively uncommon (16), and are generally associated with other types of opacities, especially in those aged >80 years old (17).

Currently, the only treatment for human cataract is surgical removal of the opaque lens and implantation of an intra-ocular lens (IOL). Cataract surgery is one of the most commonly performed elective surgical procedures in developed countries (18) and is highly successful. The main outcomes include a marked improvement in visual acuity, decreased risk of falls, and improved quality of life (19, 20). In economically developed countries, cataract blindness in the community is rare, yet across developing countries with low rates of cataract surgery, blindness from unoperated cataract is common (21). Cataract surgery is a substantial cost to global health systems. For example, in the USA, approximately 3 million surgeries are performed each year, with an estimated cost of >$3.4 billion in annual Medicare spending (19, 22). In developing countries, costs associated with cataract surgery can be prohibitive (23, 24). Hence there is a need to develop more cost-effective therapeutic alternatives to cataract surgery to delay, prevent, or reverse cataract formation (25).

Unfortunately, investigating the causes and mechanisms of human cataract formation and the ongoing effort to develop non-surgical anti-cataract therapies has associated difficulties. The use of post-mortem human donor tissue suffers from an inconsistent supply of cataractous lenses (26), as well as variable post-mortem delays between death and tissue processing (27). In addition, lenses obtained from human donors have significant biochemical variability. For example, the lifestyle, genetics, underlying diseases, and causes of death of individual donors will all contribute to this variability, and therefore the consistency of subsequent analysis. Finally, whole, cataractous lenses are now less readily available (28) due to the arrival of the extracapsular cataract surgical extraction (ECCE) method (29), where the nucleus and cortex are now emulsified and removed, leaving the capsule behind that can then be used to hold the IOL implant.

As a consequence of these challenges to utilising human tissue in cataract research, animal models have been used to investigate the underlying mechanisms of cataract formation (26) following a range of cataractous insults. While animal models of cataract aim to recapitulate the characteristics seen in human cataract that take many decades to develop, they are often induced in a laboratory environment over a relatively short time period. Lens parameters that are typically monitored in these models include transparency and morphological changes (that induce light scattering), biochemical changes (such as antioxidant depletion and pigmentation), and biomechanical changes (such as stiffening of the lens) that only manifest as cataract in later in life (30–32).

Animal models that mimic the distinctly different cataract phenotypes observed in ARN cataract (27) and diabetic cortical cataract (33) have previously been reviewed. In this review, animal models used to determine the mechanisms of lens cataract formation following exposure to UV radiation are presented and evaluated. We will first review the evidence for the cataractous effects of UV radiation in humans, and the intrinsic properties that the human eye has to protect against cataract formation. This will provide a contextualisation of the animal models used to study the role of UV exposure in cataract formation.

### 1.2 UV light in human cataract formation: exposure, epidemiology, and effects of aging

UV radiation is a known toxin to biological tissues and is classified as a carcinogen (34). The sun produces UV radiation in the UV-A, -B, and -C ranges. Approximately 97% of the wavelengths of radiation that pass through the atmosphere and reach Earth are UV-A (λ = 315-400nm), while ~3% is UV-B (λ = 280-315nm) (35, 36). Solar UV-C (λ = 200-280nm) is blocked by the Earth’s atmosphere (35) and UV-C wavelengths are produced in only a few settings on Earth, such as Arc welding.

Three main types of tissue damage can result from light exposure. While photothermal and photomechanical damage typically result from exposure to the upper end of visible and infrared light wavelengths, photochemical damage is the result of exposure to wavelengths in the UV and visible light range (37). Photochemical damage is further divided into three types. Ablation is utilised extensively in ophthalmology, where high energy wavelengths under 200 nm remove or shape ocular tissue structures. In contrast, both photo-oxidative damage and photosensitised reactions are the result of UV-A and UV-B exposure, typically as a result of long exposure times (37).

Several mechanisms have evolved to protect the eye from the phototoxic effects of UV radiation. For example, the cornea absorbs the majority of incoming UV-B light and a small amount of UV-A (38–41). However, the age of the eye has an impact on UV light penetration and consequently the amount of UV light entering the eye and reaching the lens increases with age (38). Once adulthood is reached, it is assumed that the retina is no longer exposed to UV radiation, due to the decreasing transmission properties of the lens (42). The lens absorbs most of the incoming UV-A, and the small amount of UV-B radiation that is not absorbed by the cornea (**Table 1**) (39, 40).

**Table 1:** Corneal absorbance of incoming UV light as a function of age, and lenticular absorbance of incoming light.

UV Range	λ (nm)	Cornea (% absorption)		Lens (% absorption)
		Young	Old	
**UV-B**	280-315	90 (38) – 92 (39)	60 (38)	36 – 52 (39)
**UV-A**	315-400	18 (39) – 45 (38)	80 (38)	2 (39)

Considerable epidemiological evidence shows the harmful effects of different UV wavelengths of light on the lens. The World Health Organization estimates that cataracts in up to 20% of the people who become blind annually may be caused or enhanced by sun exposure (43). Generally, UV-B light has been associated with an increased risk of cortical cataracts (**Figure 1A**) and subcapsular cataract (**Figure 1C**) (44–46), but there is less evidence for the effects of UV-B exposure on nuclear cataracts in humans (47, 48). This is possibly due to its limited depth of penetration into the lens in humans (49), monkeys (50), and rats (51). Although once dismissed as a risk factor for cataract, UV-A has since been associated with nuclear cataract formation (**Figure 1B**) (52, 53), with UV-A light shown to penetrate deep into the lens nucleus of guinea pigs (44).

Epidemiological studies have shown that higher rates of cataract are observed in populations that spend more time outdoors (54) or in the sun (55), in rural as opposed to urban living (56–58), and other specific geospatial relationships (17, 47, 48, 57, 59–73). For example, higher exposure to sunlight significantly increases the risk of age-related cataract, with a slight increased risk of cortical cataract, but no risk effect on nuclear or posterior subcapsular cataract (74). This higher exposure to sunlight can be from reflection of UV from different surfaces in the environment, with snowfields and/or increased altitude (75) having the most reflection, and forest the least (76). Interestingly, prevalence of the type of cataract appears to change with global location. Sasaki and colleagues showed that cortical opacification was more prevalent in Iceland and Japan, while nuclear cataract was more prevalent amongst Singaporeans (77). Furthermore, variations in populations within Japan show an increased prevalence for nuclear cataract formation in Okinawa due to high UV exposure (78).

Brunescence, the process of progressive pigmentation of the aging human lens which turns a young, colourless lens increasingly yellow, brown and even black, has been specifically linked to UV exposure (53). Moreover, brunescent cataracts are particularly prevalent in populations living in tropical regions of the world due to their higher exposure to solar radiation (76, 79, 80). Several of the chromophores and fluorophores (81, 82) responsible for lens colouration have been isolated and identified, including advanced glycation end products (83–86), and tryptophan oxidation products (87–89). Interestingly, some of these tryptophan metabolites are beneficial in young lenses where they play an important role in the intrinsic UV protection mechanism of the eye but become detrimental to the lens following chronic exposure to UV.

### 1.3 Lens UV exposure protection mechanisms

The young lens contains several tryptophan metabolites, which act as UV filter compounds that absorb light in the 300 to 400 nm wavelength range (90, 91). Approximately 95% of the light that enters the lens is absorbed by these compounds, with the remaining 5% being absorbed by tryptophan residues on proteins (92). UV filters also decrease chromatic aberration, thus enhancing visual acuity (93), and aid in protecting the retina from induced photo-oxidative damage (92). Synthesis of UV filters occurs between late pregnancy and birth, with some filters detectable in lenses five months post-natal (94). There are two main types of filters: primary and secondary filters. In young lenses, the ratio of primary to secondary is approximately 10:1, but this decreases with age to 2:1 (91).

**Figure 2:**
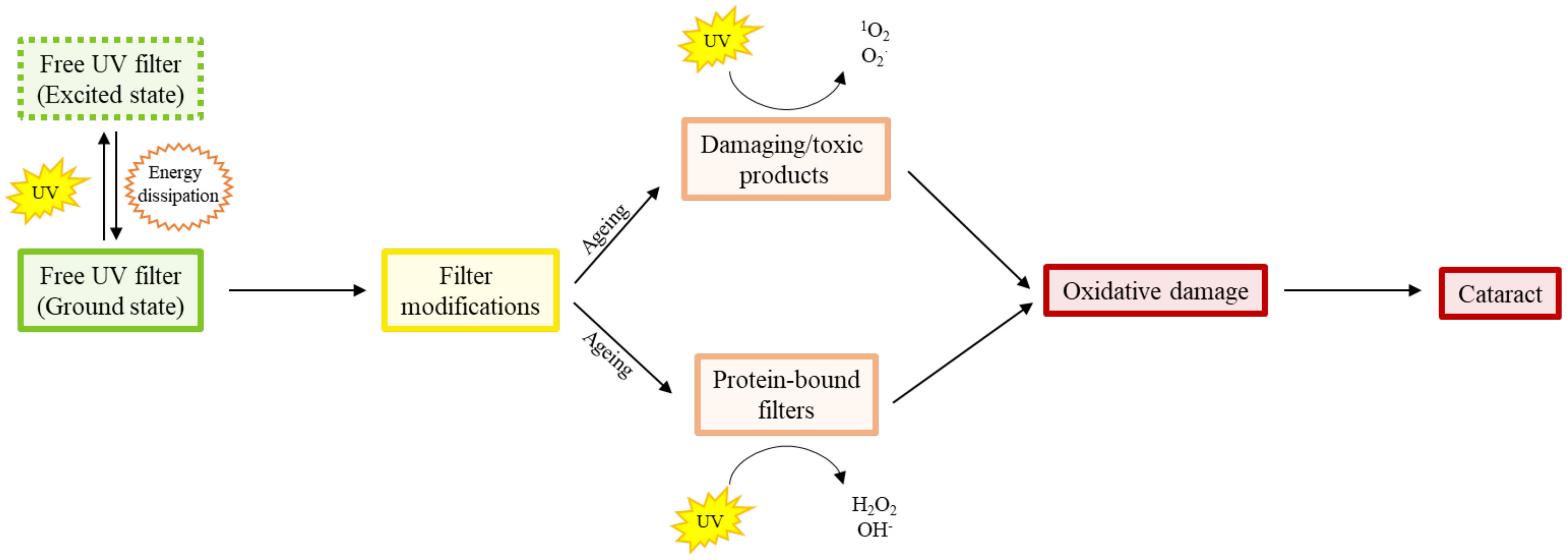
Diagram showing the age-related shift in proportion of free human UV filters to modified free and protein bound filters that produce oxidatively damaging species. The free (i.e. not bound to proteins) filters absorb UV light and dissipate the UV energy efficiently. However, with increasing age, there is conversion of filters to different compounds which produce singlet oxygen and superoxide radicals, and the binding of filters to proteins which produce peroxide and hydroxyl radicals, in response to UV light. It is this age-dependent accumulation of oxidative damage that is thought to be responsible for the initiation of ARN cataract.

When found in their free form, both primary and secondary filters are photochemically inert, and act to dissipate UV energy (95) without the production of harmful radicals (93, 96, 97), that could induce oxidative stress (97) (**Figure 2**). Photo-oxidative damage occurs when incident light reacts with a tissue chromophore such as a UV filter, which then attains an excited state. Reactive oxygen species (ROS) are generated through interaction of the excited state chromophore with a variety of substrates, which go on to oxidatively damage biomolecules (37). In contrast, photosensitisation reactions occur when oxygen and a photosensitiser molecule absorb the UV to produce hydrogen peroxide (H_2_O_2_). This can either be detoxified by the action of glutathione peroxidase, or go on to form the hydroxyl radical, which can damage a range of biomolecules, including DNA, proteins, and lipids (37).

While the young lens contains high levels of glutathione (GSH) to protect it from oxidative stress through direct neutralisation of ROS, the age-related decline in this key antioxidant makes the lens vulnerable to cataract formation. This is due to the high concentration of cell membranes in the lens, which make it vulnerable to damage from free radical-mediated lipid peroxidation (37), its high protein concentration which can form irreversible protein-protein cross-links (12), and a variety of naturally occurring small molecules, such as UV filter molecules. While UV filters are highly efficient at dissipating energy, modifications to the filters, and the binding of filters to proteins within the lens, can change their ability to quench UV radiation (95), and instead act as photosensitisers in the aging human lens (98). These filters and their modifications are discussed herein, and their classification summarised (**Table 2**).

**Table 2:** Classes of UV filters within the human lens.

Class of filter	Molecules	References
Primary	Kynurenine	(105, 263)
	3-hydroxykynurenine (3OHK)	(93, 102, 217)
	3-hydroxykynurenine glucoside (3OHKG)	(216, 266)
Secondary	GSH-kynurenine	(91, 267)
	GSH-3OHK	(267)
	GSH-3OHKG	(117, 268–270)
	Cys-3OHKG	(118)
	4(2-amino-3-hydroxyphenyl)-4-oxobutanoic acid O-β-D-glucoside	(94, 268)
	4(2-amino-3-hydroxyphenyl)-4-oxobutanoic acid O-β-D-di-glucoside	(99, 115, 267)
Modified	Kynurenine-yellow	(109)
	3OHK-yellow	(109)
	3OHKG-yellow	(109)
	3-hydroxyanthranillic acid	(102, 104)
Damaging/toxic products	N-formyl-kynurenine	(104)
	Kynurenic acid	(104, 125, 129)
	Xanthurenic acid (XA)	(95, 128)
	Xanthurenic acid glucoside (XAG)	(95, 126)
	Oxo-, dioxo-, XAG	(128)
Protein interacting	Protein-kynurenine	(96, 131)
	Protein-3OHK	(119, 134)
	3OHK crosslinked products	(110)
	Protein-3OHKG	(110)

Primary and secondary filters act to dissipate incoming UV light without the formation of reactive or damaging species. The damaging and toxic products produce singlet oxygen and superoxide when excited by UV light, and protein bound filters produce peroxide and hydroxyl radicals when excited by UV light.

The primary UV filters in the human lens are kynurenine (kyn), 3-hydroxykynurenine (3OHK), and 3-hydroxykynurenine *O*-β-D-glucoside (3OHKG) (99–102). One of the intermediates in the formation of kyn from tryptophan metabolism, N-formyl-kynurenine (NFK), differs from other tryptophan metabolites in its photophysical properties, in that it acts as a photosensitiser to produce singlet oxygen and superoxide (103, 104). In the presence of oxygen, NFK is synthesised enzymatically by indoleamine 2,3-dioxygenase (IDO), which has been found in human lenses (105), or through tryptophan photolysis following *in vitro* exposure to UV light (106). NFK has been shown to bind to crystallin proteins under oxidative stress *in vitro* (107), and during exposure to sunlight (108), suggesting that in the absence of UV filters, it could be a key mediator of UV light induced damage in the lens. 3OHKG is the most abundant filter (109), and is formed via glycosylation of 3OHK (110). Kyn, 3OHK and 3OHKG are found prominently in young lenses (102), but decline at a rate of ~12% per decade, with kyn and 3OHK being nearly undetectable in 80 year old lenses (110).

The amino acid side chain of primary filters is unstable, and is thought to be able to spontaneously deaminate, to form an α-β-ketoalkene (109, 111–113), which is also highly unstable (94). The primary filters are able to form GSH adducts, whereby a molecule of GSH scavenges the deaminated filter, potentially protecting lens proteins from covalent binding of filters (111, 114). NAD(P)H has also been identified as a protective agent, scavenging the deaminated filters (109). High concentrations of GSH, such as those in young lenses, can protect the lens in two ways: by scavenging filter deamination products, and promoting the decomposition of kyn-protein adducts. GSH-conjugated UV filters increase with a corresponding decrease in free GSH, and therefore may contribute to a decreased capacity for nuclear GSH to protect lens proteins from cross-linking and insolubilisation (110). In addition to glutathionylation, all three primary filter compounds can also undergo cyclisation to form 3OHKG-yellow, kyn-yellow, and 3OHK-yellow respectively, although this is thought to be a slow process (109). Enzymatic modification of kyn can result in the formation of kynurenic acid, which acts as a photosensitiser and produces reactive oxygen species (104).

The secondary filters 4-(2-amino-3-hydroxyphenyl)-4-oxobutanoic acid *O*-β-D-glucoside (AHBG) and glutathionyl-3-hydroxykynurenine *O*-β-D-glucoside (GSH-3OHKG) are found predominantly in the lens nucleus (98, 110, 115, 116). The α-β-ketoalkene formed through primary filter deamination undergoes reduction to form AHBG (94), binds to GSH to create GSH-3OHKG (117), or free cysteine (118), and can bind to proteins through lysine, cysteine and histidine (119). 3OHK can also form 3-hydroxyanthranilic acid (3OAA), through the enzyme kynureninase (102). This molecule is also photochemically inert and inhibits the crosslinking of crystallins within the lens (104). High levels of GSH should prevent the autooxidation of 3OAA, but with falling GSH levels in aging lens, autooxidation can occur, producing H_2_O_2_ that can damage crystallins (120). For secondary filters, AHBG can undergo additional glycosylation to create 4-(2-amino-3-hydroxyphenyl)-4-oxobutanoic acid *O*-β-D-di-glucoside (AHBGD), but neither of these filters can bind to lens proteins. This is because neither compound is able to undergo deamination, in contrast to the other filters (121).

With increasing age, the levels of free UV filters decrease markedly (110), to the point where protein-bound UV filters and free UV filters are equal in concentration in the centre of normal lenses (122). UV filters, however, are present in cataractous tissue at higher concentrations than aged-matched controls (123). Deamination of the UV filters appears to be more pronounced in the nuclear region of the lens (110). This, in combination with the age-related decrease in nuclear GSH (124), would make the nuclear region more susceptible to the covalent linkage of UV filters to crystallin proteins.

In addition to binding to proteins, UV filters also create some damaging products. Xanthurenic acid (XA) is proposed to be one of the damaging products created through filter modification, although there are conflicting findings on whether or not XA is present in normal human lenses (103). However, it is present in cataractous lenses (125), with its glucoside (XAG), being present in brunescent cataracts (95, 126). XA could be formed enzymatically within the lens, from 3OHK, or through oxidation of 3OHK-yellow (127, 128), or through 3OHKG (95, 129). In addition to its glucoside, XA can be oxidised to form oxo-xanthurenic acid (OXA) and subsequently dioxo-xanthurenic acid (DOXA). DOXA may induce oxidative stress by generating oxygen free radicals, and also denature proteins through the crosslinking of crystallin proteins within the lens (128).

It is hypothesised that instead of protecting the lens from oxidative damage, the protein-bound UV filters may initiate oxidative damage, or act as an oxidant (130), resulting in the formation of proteins with altered physical and chemical properties (96, 98). These alterations include cross-linking, oxidation, fragmentation, peroxide formation, amino acid isomerisation, unfolding, and alterations to protein solubility (96–98, 131–134). The coloration or brunescence seen in the cataractous lenses is thought to be a result of accumulated oxidative reactions involving protein bound UV filters (121).

In summary, it has been shown that the human lens has developed a collection of filters to absorb the UV-A and UV-B light that passes through the cornea and penetrates into the different regions of the lens. UV light causes the degradation of these filters, with GSH preventing some of these damaged filters from binding to proteins. With age, the amount of UV light reaching the lens increases as the UV filtering capacity of the cornea declines. This increase in the incidence of UV light, plus a reduction in the efficacy of the filters and a parallel age-related decrease in the GSH availability in the lens, produces oxidative stress that leads to cataract formation. In the next sections, we review what we have learnt about the effects of UV light on lens transparency from a variety of different animal models and critically assess whether these models accurately model the effects of UV exposure seen in the human lens.

### 1.4 The use of animal models to mimic UV-induced cataract in humans

To understand how UV radiation induces lens cataract, a considerable number of studies have exposed animal lenses, either *in vivo* or *ex vivo*, to UV light (**Supplementary Table 1**). For *in vivo* models, sub-threshold doses can be applied over many days as cumulative, chronic doses, whereas *ex vivo* models are subject to tissue degradation, and therefore often use acute, super-threshold doses. While *in vivo* models can better mimic the processes that occur in a whole system than an *ex vivo* lens, this comes at added time and financial cost. In addition, the penetration of UV light through the cornea changes depending on the animal model used. Hence, *ex vivo* models that use the lens alone must also consider that the dose given to the lens may be different to what the lens would experience *in vivo*, due to the lack of protection from the cornea. While both *in vivo* and *ex vivo* models can be used to assess recovery of lens tissue post-exposure, *ex vivo* models are again constrained by tissue degradation and time post-mortem. Despite these limitations, *ex vivo* models can be exposed to large doses of UV without concerns for animal welfare. In addition, lenses from larger animals, such as pigs and cows, can often be obtained as a by-product from abattoirs and are more cost effective than tissue derived from smaller laboratory animals. The downside of this, however, is that the exact age and other potential confounding factors such as disease, sex, and post-mortem time is less precise than small laboratory animals sourced in-house.

Despite the above factors, both *in vivo* and *ex vivo* models have been very effective in elucidating the mechanisms underlying cataract initiation and progression following UV exposure (**Table 3**). However, the relevance of the chosen animal model to the level of exposure and cataract development in the human lens is often not critically assessed. In each section of this review, we have assessed the relative merits of the existing animal models of UV cataract and have assigned the models to one of two categories: 1) Nocturnal animal models where “non-environmental” UV exposure serves as an oxidative stress that compromises lens transparency, and 2) UV light exposure in crepuscular and diurnal animals that could act as more relevant models that mimic the effects of UV light on cataract development in humans. While many of these animal studies investigated alterations in gene expression (135–140) and DNA damage (141, 142) upon irradiation, in this review we focus on the morphological, biochemical, metabolic, and protein changes that characterise the cataract phenotype induced as a result of UV-A or UV-B exposure.

**Table 3:** Key features of UV cataract in humans - a comparison to animal models of UV exposure.

		Antioxidants			Protein changes			Impaired enzymatic function	Lens colouration	Cataract phenotype	
		Total	COR	NUC	↑ mixed disulfide	↑ WI fraction	Aggregation			COR	NUC
Human	*In vivo*	↓	NC	↓	✓	✓	✓	✓	✓	✓	✓
Mouse	*In vivo*	↓ ↓	**- -**	**- -**	✓ ✓	**- -**	**- -**	✓ ✓	**- -**	✓ ✓	✓ **-**
Rat	*In vivo*	**- -**	**- -**	**- -**	**- -**	**- -**	**- -**	x ✓	**- -**	✓ **-**	✓ **-**
	*Ex vivo*	**- -**	**- -**	**- -**	**- -**	**- -**	**- -**	✓ **-**	**- -**	✓ **-**	**- -**
Guinea pig	*In vivo*	- ↓	- NC	- ↓	- ✓	- ✓	- ✓	**- -**	- ✓	- ✓	- ✓
	*Ex vivo*	**- -**	**- -**	**- -**	- ✓	**- -**	**- -**	**- -**	**- -**	✓ **-**	**- -**
Rabbit	*In vivo*	↓ **-**	**- -**	**- -**	**- -**	**- -**	**- -**	**- -**	- ✓	✓ x	x x
	*Ex vivo*	**- -**	**- -**	**- -**	**- -**	**- -**	**- -**	**- -**	**- -**	✓ x	**- -**
Cow	*Ex vivo*	**- -**	**- -**	**- -**	**- -**	**- -**	- ✓	- ✓	**- -**	✓ ✓	**- -**
Pig	*Ex vivo*	**- -**	**- -**	**- -**	**- -**	**- -**	**- -**	**- -**	**- -**	✓ ✓	**- -**
Squirrel	*In vivo*	**- -**	**- -**	**- -**	- ✓	- ✓	- ✓	**- -**	**- -**	**- -**	**- -**
	*Ex vivo*	↓ **-**	**- -**	**- -**	**- -**	**- -**	- ✓	**- -**	**- -**	✓ ✓	- ✓

Blue symbols = UV-B, Green symbols = UV-A, COR, cortex; NUC, nucleus; ↓, decrease; -, not reported; ✓, present; x, absent; NC, no change.

## 2 Nocturnal animal models of UV as an oxidative stress

Due to their size and ease of housing, mice and rats have proven to be popular choices for the development of models of UV cataract formation. However, the most widely used rodent animal models are nocturnal and not naturally exposed to the high levels of UV light experienced by diurnal animals. Moreover, rodent models are often exposed to UV light at much higher doses than diurnal animals experience environmentally in order to shorten the experimental time course required for the development of cataract. Due to their low natural exposure to UV radiation nocturnal animals do not express the same system of UV filters seen in the human lens. Therefore, the same radiation energy dissipation that occurs in the human lens does not occur in mice and rat lenses that do not have UV filters.

### 2.1 Mice

Mice have been used as models for many types of cataract (see (27)). Important differences between the mouse and human lens include a different distribution of β-crystallins (143) and crystallin proteins that are modified differently (144). Critically, however, mice see in the ultraviolet range (145), and thus their lenses contain no UV filters to absorb UV radiation (32). Despite these differences, mice have been used to study the effects of both UV-A and UV-B radiation on lens protein content, as well as the morphological and biochemical status of UV-exposed lens. Murine tissue has also been used to assess the efficacy of external agents, such as caffeine and ascorbate, in preventing UV cataract *in vivo* (146, 147).

#### 2.1.1 Cataract phenotypes induced by UV light exposure

To establish the relative toxicity of UV light exposure to lenses, mice have been exposed to UV-A or UV-B for up to 39 weeks. UV-A exposure was found to be weakly cataractogenic when compared to UV-B in albino mice *in vivo* (148, 149). *In vivo* exposure of mice to UV-B has not only induced subcapsular cataract, but also cortical and nuclear cataract (150). Further development of this model showed that when only one eye was exposed to UV-B, the non-exposed eye suffered intraocular inflammation and an increase in lens light scattering also, perhaps due to a co-cataractogenic inflammatory response (151).

#### 2.1.2 Effects of UV light on metabolism, antioxidant pathways and protein function

Changes to lenticular protein concentration of albino mice in response to UV-A has been investigated *in vivo* (152, 153). Following long-term exposure to UV-A (up to 87 weeks), insoluble protein levels rose to 46% higher than controls (153), which is similar to the accumulation of insoluble proteins observed in ARN cataracts in humans (124, 154). Subcapsular and cortical opacities were observed between 30 and 50 weeks, after which anterior cortical cloudiness was observed. While the cataract phenotype observed here was different to that observed in humans, these results confirmed that long-term *in vivo* exposure to UV-A light leads to cataract in an albino mouse model.

Morphological and biochemical alterations produced as a result of *in vivo* exposure of mice to UV-B radiation have been investigated (155). Within two days of exposure, the mice had developed anterior subcapsular cataracts, similar to results from another study (156), with the onset of morphological changes beginning at 24 hours post-exposure. Importantly, older mice showed more prominent macroscopic changes compared to younger mice, and GSH depletion was more prominent in the older lenses than the younger lenses, again reflecting changes observed in human lenses. Glyceraldehyde-3-phosphate dehydrogenase (G3PD) inactivation was more exaggerated in the older lenses, diminishing ATP production and having a direct impact on lens transparency.

In addition, the enzymes thioltransferase (TTase) and thioredoxin (Trx) were upregulated following UV exposure, likely providing oxidative damage repair in the younger mice. Trx has been shown to play an important role in defending against UV-A light in cultured human epithelial cells (157). The decrease in G3PD function, which can be restored by dethiolation of TTase, was thought to be a result of suppressed enzyme activity by UV exposure, rather than a direct effect on protein expression levels. While there are differences in how deep UV will penetrate into the mouse eye versus the human eye, the same age-related deterioration in enzyme function is seen in humans (158), suggesting that a similar response to UV-B exposure may occur in the human lens.

In summary, while prolonged UV-A exposure to mice *in vivo* induces a variety of cataract morphologies that differ to those observed in humans, these studies showed that elevated protein insolubility and impaired enzyme function are caused by UV-B irradiation. In addition, older lens tissue appears to have a reduced capacity for repair compared to younger lens tissue.

### 2.2 Rats

Rats are one of the most commonly used laboratory animals. However in comparison to human lenses, rat lenses have different protein distributions (159). Relative to body size, rats have larger lenses and thinner corneas than humans (49, 160). Rat corneas attenuate less UV-B and more UV-A radiation than the human cornea (49). Rat lenses also do not accommodate, due to poorly developed ciliary muscles (161). In addition, rat lenses transmit almost all incoming UV-A, which can damage the rat retina (162), suggesting that rats lack UV-A absorbing compounds (32). Despite these fundamental difference to human lenses, numerous studies have investigated the effects of UV light exposure on lens morphology and biochemistry.

#### 2.2.1 Cataract phenotypes induced by UV light exposure

Acute exposure of rat lenses *in vivo* to both UV-A and UV-B cause a variety of cataract phenotypes (163, 164). Results from these studies have suggested that the lens epithelium exhibits an ability for regenerative repair, which is not observed in cortical fibre cells. Dose accumulation of UV-B radiation was assessed in a chronic exposure rat lens model (160). Lenses that were exposed to UV irradiation developed cataracts on the anterior surface. In addition, the anterior lens opacities intensified in all exposure period groups with the increasing cumulative dose. However, the sensitivity of the lens to UV-B radiation decreased with the number of days during which the dose was accumulated, suggesting that repeated exposure to UV-B decreases the lenses ability to recover and repair damage (160).

#### 2.2.2 Effects of UV light on metabolism, antioxidant pathways and protein function

The localised cell swelling induced in the anterior surface of the rat lens induced by *in vitro* UV-A exposure appears to be due to an effect of UV-A on Na^+^/K^+^ ATPase activity, which decreased in both the lens epithelium and cortex (164). Low Na^+^/K^+^ ATPase activity may also underly cataract formation following exposure to UV-B, where lactate dehydrogenase (LDH) activity, and therefore ATP production, was lower predominantly in the anterior lens regions (epithelium and outer cortex), which was consistent with the pattern of exposure (51). While the decrease was relatively small (20%), this suggested a role for decreased ATPase activity in UV-B-induced cataract formation. However, this mechanism is yet to be confirmed in human lenses exposed to UV-B.

Changes in the metabolic profile of lenses exposed to UV-B radiation may also be anticipated if ATP production is affected as shown by Löfgren and Söderberg (51). Decreases in phenylalanine, GSH, and succinate, have been detected, potentially due to their leakage from the lens following membrane damage from UV irradiation (165). Metabolite levels can be restored following UV-B exposure, although the timeframe of metabolite decrease and restoration can vary (166). It would be interesting to apply this approach to study UV-B exposure in human lenses, and whether similar changes to metabolites and LDH are observed, whether metabolite levels can be restored, and whether this exposure would produce similar cataract phenotypes to those observed in the rat.

The effect of UV-B irradiation on lens glycolysis has been investigated in Sprague-Dawley rats (167). Lactate (produced by LDH) is an end product of anaerobic glycolysis and is often used as an indicator for activity of the glycolytic pathway (168). Lactate production was reduced initially, however, six hours after exposure, the lactate level in the exposed lenses was greater than contralateral lenses (167).

*In vitro*, irradiation of both intact and homogenised rat lenses has shown decreased activity of enzymes involved in the major metabolic pathways. For example, hexokinase, G6PD, aldose reductase, and Na^+^/K^+^ ATPase showed decreased activity of up to 57% compared to the control lenses, although UV-B exposure did not result in cataract formation (169). Interestingly, physiologically relevant levels of antioxidants (vitamins C and E, and β-carotene) that were added to the lens incubation medium during irradiation prevented the perturbation of enzymatic activity detected in UV-B-exposed lenses in a concentration dependent manner (169), suggesting that the damage to enzymes was through an oxidative stress mechanism. While enzyme activity changes may be involved in UV-B-induced lens opacity, the therapeutic potential of antioxidant supplementation for human lenses remains unclear since it has already been shown that both vitamin C and E have little to no effect on the prevention of human ARN cataracts when consumed as a dietary supplement (170–172).

Interestingly, albino rats are more sensitive to UV-B radiation than pigmented rats *in vivo* (173), possibly due to differences in corneal, aqueous humour, or iris transmission of light. The same trend is also seen with *in vitro* irradiation of lenses extracted from albino and pigmented rats (174). However, it is difficult to say whether the pigmented or albino rat is more suitable as a model for UV cataract.

In summary, rat models have shown that both UV-A and UV-B can impact cation homeostasis through Na^+^/K^+^ ATPase, and both ranges of wavelengths can create anterior subcapsular cataracts in the rat lens. The rat lens has been shown to be most susceptible to UV-B at 300 nm (175), with most of these models employing this wavelength. UV-B increases light scattering, and also decreases water soluble metabolites, enzyme activities, and cellular respiration.

## 3 Crepuscular animal models of human age-related cataract

Crepuscular animals are active during twilight hours of dawn and dusk. The UV index at twilight is approximately 200 µJ cm^-2^ (176), and therefore considerably less than the average 2 J cm^-2^ experienced during the day. However, the dose of UV that these animals would be exposed to in their natural environment is more than nocturnal animals and more similar to humans. Thus, crepuscular animals have been used as animal models of human age-related cataract. Crepuscular animals can be used *in vivo* as they are relatively small and easy to keep, and *ex vivo* tissue is readily available.

### 3.1 Guinea pigs

Guinea pigs have previously been used for models of ARN cataracts (177–180), and galactose-induced cataract (181, 182). Due to high levels of a UV-A chromophore in the lens (122) that mimics that of the human UV filter, a similar pattern of lens GSH distribution with lower levels of GSH in the nucleus relative to the cortex (183), and a similar age-dependent pattern of nuclear disulfide formation (184), it is proposed that guinea pigs are the best non-primate model currently available for the study of UV-A exposure and cataract (32).

However, while there are many benefits to the use of guinea pigs as a model of human UV exposure, it is also important to consider inconsistencies between humans and guinea pigs. For example, there is conflicting evidence for the formation of brunescence in the guinea pig lens (185, 186), unlike the characteristic time dependent increase in colouration observed in the human lens (187).

#### 3.1.1 Cataract phenotypes induced by UV light

Chronic exposure of guinea pigs to a low level of UV-A light *in vivo* produces protein aggregation and cataract in the centre of the lens (188). In contrast, the anterior lens cortex showed no difference between UV-A exposed and control guinea pigs, perhaps due to the higher metabolic activity, and therefore antioxidant capacity of the lens cortex. The mechanism of nuclear cataract formation in the guinea pig nucleus may be due to the binding of the UV-A chromophore NADPH to zeta crystallin. This is proposed to mimic the binding of kynurenine to crystallin proteins in the human lens (122). NADPH is known to cause the formation of superoxide ions (189), and H_2_O_2_ (190), when it absorbs UV-A (see **Figure 2**). Interestingly, the guinea pig is over 10-fold more tolerant to UV-B than pigmented rabbits, rats, and mouse (191). Very high doses of UV-B are required to produce cortical cataracts in the anterior subcapsular region of the lens *in vivo*. The exact mechanism for this enhanced protection is unclear, although perhaps it is because the guinea pig lens contains more ascorbate than rat lenses, with levels similar to the human lens (191), as well as high levels of free NADPH (44, 192) and zeta-crystallins (193).

#### 3.1.2 Effects of UV light on metabolism, antioxidant pathways and protein function

Interestingly, UV-A light produced deleterious effects on the nucleus of guinea pig lenses, when compared to age-matched controls (44). There was an increase in light scattering, distention of intracellular spaces, a decrease in GSH, increased lipid peroxidation, and a loss of water-soluble proteins in the lens nucleus. Results from UV-A exposure of guinea pig lenses *in vivo* strongly support the role of oxidative stress in cataract formation following UV exposure. For example, lenses show up to a 50% reduction in free sulfhydryl, with a concomitant 100% increase in disulphide formation (185). This is possibly due to the formation of protein mixed disulphides (44), or reduction in activity of glutathione reductase, such as that suggested from studies of squirrel lenses (194). The guinea pig has also been used to test if ascorbate delivered by the diet (195–197) can protect against photooxidative damage to the lens induced by UV exposure. While this work showed promise, more recent investigations showed that ascorbate does not prevent human ARN cataract formation when consumed as a dietary supplement (170, 171).

In summary, *in vivo* guinea pig models show that UV-A can penetrate deep into the lens nucleus and cause dense nuclear opacification, as well as brunescence of lens tissue. In addition, ROS may be generated, which may contribute to changes to the lens after exposure that are characteristic of oxidative stress. Nuclear opacification, protein aggregation, loss of free GSH, and increased levels of disulphides within UV-A exposed lenses show how potentially damaging UV-A radiation can be *in vivo*. Guinea pigs appear to be quite tolerant to UV-B radiation, with very high doses required to produce cataracts.

### 3.2 Rabbits

Rabbits have previously been used as *ex vivo* models for ARN cataract formation (198) and oxygen-induced protein changes (199, 200). Cultured rabbit lens epithelial cells have also been used to investigate the effects of UV-A and/or UV-B (201–206), as well as the efficacy of UV blocking contact lenses (207, 208). While rabbit lenses lack kynurenine-based UV filters (32), they contain high levels of NADH and NADPH (207) which absorb UV-A light and may therefore act as a human UV filter analogue. Rabbit lenses are also more similar in size and sphericity to human lenses than other commonly used rodent lenses and have been used in both *ex vivo* and *in vivo* studies.

#### 3.2.1 Cataract phenotypes induced by UV light

The rabbit cornea absorbs radiation completely at wavelengths at, and below 290 nm (209), and therefore the lens is more susceptible to damage at wavelengths greater than 290 nm. UV-A irradiation of rabbit lenses (210) produced opacification, potentially due to the tight bundling of actin filaments, or other morphological effects indicating cell cytotoxicity, including the breakdown of plasma membranes. Relatively low exposures of UV-B can produce anterior subcapsular lenticular opacities *in vivo*, although these opacities are not permanent and resolve within three months (209).

After *in vivo* irradiation of rabbit lenses, the lenses exhibit a pale yellow colour, although the reason for this colouration is unclear (207). Given that human lenses show colouration with aging, this is an interesting finding and suggests the potential of the rabbit lens for investigating the effects of UV light on lens colouration, despite differences in UV absorbing compounds.

#### 3.2.2 Effects of UV light on metabolism, antioxidant pathways and protein function

Changes to the metabolic profile of *in vivo* albino rabbit lenses with either a single dose, or repeated exposures adding up to a single dose, of UV-B has been investigated (211). Interestingly, repeated exposure to a small dose had more of an impact on the lenticular metabolic profile than a single dose (equal to cumulative repeated dose) did, demonstrating the cumulative effect of repeated UV-B irradiations. No lenticular opacification was reported, therefore, combined with the results of Pitts (209), it is unclear whether UV-B irradiation produces permanent cataracts in rabbit lenses *in vivo*. In contrast, exposure of *ex vivo* albino rabbit lenses to UV-B does produce lens opacification. This appears to confirm that impairment of Na^+^/K^+^ ATPase function is a common mechanism for UV-induced lens cataract (212), while highlighting that *in vivo* and *ex vivo* exposure systems can produce conflicting results.

Cultured lenses from rabbits four to five weeks old are not sensitive to UV-A irradiation alone (213), which contradicts previous findings (210), although the age of the rabbit may influence the extent of UV-A sensitivity. However, a combination of UV-A and UV-B has been shown to be more damaging to the cultured rabbit lens than UV-A alone (213). *In vivo* exposure to combinations of UV-A and UV-B using a variety of protocols have not consistently produced changes to rabbit lens metabolites (211, 214), proteins or malondialdehyde as a marker of oxidative stress (215). Taken together it appears that the rabbit lens *in vivo* is relatively well protected from UV-induced damage.

## 4 Diurnal animal models of human age-related cataract

Diurnal animals are active during the day, and sleep at night and therefore more closely resemble the activity of the average human than nocturnal and crepuscular animals. Moreover, UV filters have been identified in diurnal animal lenses. Like humans, primate lenses have been found to contain 3OHKG (216–218). 3OHKG is the main absorbing species in young primate lenses, and both UV-A and UV-B have been shown to penetrate, and be absorbed by, the young lens nucleus (219). Primate lenses have similar optical and biometric properties to human lenses (220), but tissue can be difficult to obtain. Primate lenses have been used to investigate changes in UV absorption and transmission with age (219, 221), and effects of UV radiation on the cornea (222). However, to our knowledge, primate lenses have not been used experimentally to assess the impact of UV radiation on the lens, and thus will not be discussed further. Grey squirrels (223, 224), thirteen-lined ground squirrels (225, 226), cows (227), fish (228–230), and other mammalian lenses (231), have been shown to contain tryptophan metabolites or UV sensitive pigments. While smaller diurnal animals (e.g. squirrels) can be studied *in vivo*, most diurnal animal models generally use *ex vivo* tissue.

### 4.1 Cows

Bovine lenses are thought to express limited UV filter compounds, such as 3OHK (227), but share a similar predominance of α-crystallin (232, 233). Bovine lenses also do not undergo significant accommodation (234). There are UV models that utilise isolated bovine proteins (235–238), and epithelial cell cultures (239), however they will not be discussed.

#### 4.1.1 Effect of UV light on cataract phenotype

Daily exposure of *ex vivo* bovine lenses to UV-A results in no significant changes to transmittance or focal length, when compared to controls (240). Mild subcapsular opacity is also observed. When *ex vivo* bovine lenses were exposed to UV-B, the results show that weekly doses of UV-B prevented lens repair, but these changes should not be considered to be cumulative, since the damage did not worsen with subsequent doses (240). 

The effect of varying low-level UV-B exposure on light scattering and lens focal length *ex vivo* has also been investigated (241). Although not statistically significant, the second lowest dose (0.06 J cm^-2^) appeared to have the greatest impact on the measurement parameters. Slight anterior subcapsular opacities became apparent as soon as two hours after irradiation, but in most cases, the damage cleared and only a very small area of damage remained on the anterior surface of the lens. Most of the exposed lenses showed measurable increases in light scatter and focal length but were able to recover. Thus, low doses of UV-B radiation do not permanently damage *ex vivo* bovine lenses, similar to findings in rabbit lenses (209).

#### 4.1.2 Effects of UV light on metabolism, antioxidant pathways and protein function

The effect of UV-A irradiation and subsequent recovery on the biochemical and optical properties of *ex vivo* bovine eyes has been investigated (242). The activity of hexokinase, catalase, and G6PD enzymes is perturbed by UV-A in a dose-dependent manner. Hexokinase appears to be the most sensitive to UV-A exposure, similar to the observation for rat lens hexokinase in response to UV-B radiation (243). In addition, the activity of Na^+^/K^+^ ATPase in lens epithelial cells is impaired in response to UV-A exposure (244). However, repair mechanisms exist within the bovine lens that remain intact *ex vivo*, which were able to repair damage done to optical quality and Na^+^/K^+^ ATPase activity in the central region of the lens epithelium.

The effect of UV-A irradiation on the chaperone-like properties of α-crystallin has been investigated in bovine lenses, showing differences between the response of α-crystallin from young, and old lenses (245). In comparison to young lenses, α-crystallin from old lenses had a decreased ability to inhibit protein denaturation *in vitro*. There was an increase in the molecular weight of α-crystallin fractions, and a loss of tryptophan fluorescence which barely recovered following irradiation. This suggested that older lens proteins are more susceptible to damage from irradiation, which has also been observed in mice exposed to UV-B (155). This is perhaps due to a cumulative effect of UV-A radiation, and potentially similar to the UV-B effects observed in rats (160), and rabbits (211). When the lenses began to recover (indicated by focal length repair), chaperone-like activity recovered and tryptophan fluorescence increased predominantly in young lenses, suggesting that conformational changes to α-crystallin which had occurred during irradiation had resolved.

In summary, UV-A irradiation can cause anterior subcapsular opacities in the bovine lens and older lens proteins appear to be more susceptible to UV-A damage. Permanent damage to lenticular enzyme activities can occur with sufficiently high doses of UV-A and may be implicated in UV-A induced cataract. UV-B can induce small subcapsular opacities in the bovine lens, although low doses do not appear to permanently damage the lens. However, lens repair may be prevented with repeated UV-B exposure.

### 4.2 Pigs

In comparison to humans, pigs have a similar lens protein concentration (32, 246), lens shape (247, 248), light transmission (249), and protein content (250). While pig lenses are thought not to accommodate (251), and differences in the UV filter composition of pigs and humans exist (252), some inferences on human lens response to UV radiation may be made from porcine studies. While the impact of UV-A light on protein isolates (253, 254), and lens tissue sections (255) have been investigated, we will only focus on changes induced in *ex vivo* organ cultured lenses.

#### 4.2.1 Cataract phenotypes induced by UV light

The effectiveness of different wavelengths of light has been assessed using *ex vivo* porcine lenses (256). Mid-range UV-B (295 nm) was 25 times more effective than tail-end UV-B (315 nm) radiation at producing anterior subcapsular lesions. To assess for the ability of porcine lenses to recover from exposure to UV, lenses were exposed to five times UV-B threshold exposure, resulting in the appearance of the lens sutures, suggesting the radiation had inflicted permanent damage to the lens. At two times threshold for UV-A, there was no full recovery of the lens, confirming that UV-A is cataractogenic in the porcine lens. This study concluded that the most damaging wavelengths are 270 to 315 nm, due to the low dosages required to produce visible damage. Without the protection provided by the cornea *in vivo*, the UV-B radiation was able to have a substantial impact on the lens tissue. Wavelengths shorter than 285 nm would be expected to be more damaging, since shorter wavelength photons are also higher energy (257), but this was not the case.

In addition, the same group utilised *ex vivo* porcine lenses to investigate the effect of a combination exposure of both UV-A and UV-B radiation (252). This model demonstrated the synergistic effects of low, subsolar UV-A and UV-B, with significant inhibition of cellular metabolic activity and no indication of recovery. Some recovery of plasma membrane damage was observed; however, optical quality did not recover in the study period. UV-A radiation alone required high doses (λ = 365 nm, 86 J/cm^2^) to produce significant decreases in cellular and optical integrity, in accordance with the previous study (256).

In summary, there are numerous similarities between porcine and human lenses, making them a more popular animal model of choice in recent UV exposure investigations. High doses of UV-A are required to produce anterior subcapsular opacities in porcine lenses, compared to the lower doses of UV-B required to produce the same phenotype. However, a combination of UV-A and UV-B incurs significant damage to the cellular metabolic activity and optical quality of the lens. Further experiments using porcine lenses could monitor changes to the cellular systems that are known to be involved in UV-induced damage which have been established in other models, and to investigate whether porcine lenses would become brunescent with age and/or UV exposure.

### 4.3 Squirrels

Squirrel lenses share several features in common with humans, suggesting they may be a good model animal for understanding the effects of UV exposure on lens transparency. For example, they contain UV filters (226) which have a similar structure and concentration to those found in humans (225), and display brunescence (224, 225). Squirrels have similar levels of GSH in the nucleus as a young human lens (225), but the total GSH is approximately twice that of humans (258). While cultured squirrel lens epithelial cells have been used to study the use of vitamin E as a protective agent against UV-induced damage (202, 205), only *in vivo* and *ex vivo* experiments that utilised whole squirrel lenses will be discussed here.

#### 4.3.1 Cataract phenotypes induced by UV light

The effects of ambient exposure to UV-A have been investigated in grey squirrels *in vivo* (194). Following chronic UV-A (λ = 365nm), well defined lens opacities (cortical and subcapsular) were observed, and histological analysis showed swelling of the superficial cortical fibre cells and some degenerating fibres post UV-A exposure. Anterior opacities that increase in severity with exposure time have also been observed using *ex vivo* squirrel lenses (259). The type of cataract formed from exposure to UV-B is not documented, but UV-B exposed lenses have been used for biochemical analyses (260).

#### 4.3.2 Effects of UV light on metabolism, antioxidant pathways and protein function

UV-A exposure was associated with an increase in crosslinking and degradation of crystallin proteins, and small changes in the levels of soluble crystallin proteins (194). A major loss of GSH in the outer and inner cortex was detected, while levels in the nucleus remained the same, which is opposite to the pattern seen in the aging human lens (183). Although apparent in the lens cortex, this study showed that chronic exposure of UV-A light can induce cataract formation.

Furthermore, UV-A exposure causes significant damage to phosphorous metabolites, such as ATP, in the *ex vivo* squirrel lens (259). Changes to ATP levels appear to scale with dose, whereby a lower dose causes a smaller decrease in ATP. Crystallin proteins have been shown to undergo crosslinking when exposed to UV-A in the squirrel lens (261). *In vivo*, increases in proteins with greater molecular weights occurred in the outer layers of the lens, but not the nucleus (261). This is similar to the pattern of altered lens protein distribution in cataractous human lenses (262). Crosslinking of soluble crystallins was seen in both *in vivo* and *ex vivo* exposed lenses. A link was also made between squirrel lens pigment and protein crosslinking, indicating that lens pigment stimulates the photosensitised crosslinking of lens proteins *in vitro* (261) which may provide some insight into the protein changes that occur during human cataract formation.

Indoleamine 2,3-dioxygenase (IDO) is an antioxidant enzyme, and the first-rate limiting enzyme of tryptophan catabolism. Exposure of squirrel lenses to UV-B led to an increase in IDO activity within the lens, and thus an increase in tryptophan metabolites (i.e. kyn and 3OHK) (260). Irradiation also led to increased lipid peroxidation and a decrease in GSH, suggesting UV-B had caused oxidative stress within the lens. Long durations of UV-B exposure had a small but suppressive effect on the activity of superoxide dismutase (SOD), an antioxidant protein that reduces intracellular levels of superoxide radicals. Human lenses rely on IDO for the formation of UV filters (105, 263, 264), and these findings in squirrel lenses may have parallels in human lenses.

In summary, squirrel models have been used to show the damaging effect UV-A has on proteins, initiating crosslinking and degradation. Although it is unclear if UV-B induces opacification of squirrel lenses, it does cause oxidative stress, shown through increasing lipid peroxidation and a reduction in antioxidant.

## 5 Conclusions

The paucity and significant biochemical variability of human lens tissue necessitates the use of animal tissue to model and characterise the effect that UV light has on tissue transparency and its role in cataract formation. While conclusions drawn from animal studies cannot always be directly translated to human cataract due to the morphological and biochemical differences between species, animal models have revealed several changes that take place in lenses exposed to different wavelengths of UV light with both UV-A and UV-B light appearing to play a role in cataract formation, albeit by different mechanisms. It remains clear that the same wavelength and dose of UV-A or UV-B can produce an array of different biochemical and metabolic changes, as well as cataract phenotypes, and is dependent on the animal model used. Similarly, different cataract phenotypes can result from the same underlying mechanism. While mice and rats are convenient laboratory animal models, the fact that they are nocturnal animals that normally experience completely different UV exposure levels to humans means that the role of UV light exposure in cataract development in rodents must be carefully interpreted with respect to cataract formation in the human lens. Nevertheless, these models have helped to identify oxidative stress via photooxidation, and photosensitisation as major factors involved in UV cataract development. Specifically, the Na/K ATPase activity is impaired in several UV exposure models, which is likely to impair the specialised transport system known to maintain lens tissue transparency (265).

Although crepuscular animals experience higher levels of UV radiation than nocturnal animals, they still do not experience the same levels of exposure as humans. In addition, both classes of animal lack the same range of UV filters found in the lens. However, in both types of animals the application of “non-environmental” UV exposures to these laboratory animals does provide information on UV-A and UV-B as cataractous stressors that can differentially activate oxidative defence pathways in different regions of the lens that normally act to maintain lens structure and function. Guinea pigs, however, appear to recapitulate many of the characteristics of UV cataract in humans, including lens brunescence.

Diurnal animals share more similarities with humans than nocturnal or crepuscular animals with respect to UV exposure levels, the presence of UV filters, and the characteristic brunescence of lens tissue at least in the case of the squirrel lens (225). This suggests that mechanisms of UV damage observed in squirrel lenses could be directly applicable to UV cataract formation in humans. However, not all laboratories have access to these animals.

In contrast, porcine and bovine lenses are more readily available and easily utilised in organ culture experiments. The use of these diurnal *ex vivo* animal models has shown that UV-A radiation has the potential to be more harmful than UV-B radiation, possibly due to the absorption of this longer wavelength energy in deeper lens cell layers that inactivates enzymatic activity involved in the protection against oxidative stress. They have also shown that in younger lenses at low doses this UV induced damage can be repaired; but that older lenses are more susceptible to UV damage and showed impaired recovery compared to young lenses receiving the same dose. The lack of evidence surrounding yellowing of the lens tissue with age (or UV exposure) in these larger animals, however, means that while they are useful for understanding potential changes to proteins resulting from photooxidation and photosensitisation, they lack a key characteristic of human UV cataract.

Despite this substantial body of work, gaps in our understanding of the extent of the impact that exposure to UV light has on the lens remain. To close this gap continued development of UV light exposure models that utilise diurnal animals, especially guinea pig and squirrel lenses, will further enhance our understanding of the role that UV light exposure plays in the development of human lens opacities. In addition, models that combine stressors, for example oxidative and photooxidative stress, may prove useful to further investigate human cataract development.
