# Supplementary material for: UV light and the ocular lens: a review of exposure models and resulting biomolecular changes
Source: Front Ophthalmol (Lausanne). 2024 Sep 5;4:1414483. doi: 10.3389/fopht.2024.1414483 (PMC11410779; doi:10.3389/fopht.2024.1414483)
Supplement: Supplementary file 1 [file Table1.docx]

Supplementary Material

**UV light and the ocular lens: A review of exposure models and resulting biomolecular changes**

**Emily R MacFarlane, Paul J Donaldson, Angus C Grey***

*** Correspondence:** Angus C Grey: ac.grey@auckland.ac.nz

**Supplementary Table 1. Summary of the animal models, wavelengths, and doses, in this review.**

| Animal | UV-A wavelength (nm) | Dose | UV-B wavelength (nm) | Dose | Author |
| --- | --- | --- | --- | --- | --- |
| Mouse in vivo | 320-400 | 100 µW cm^-2^ | 300 | 100 µW cm^-2^ | (1) |
|  | 320-400 | 100 µW cm^-2^ | 300 | 100 µW cm^-2^ | (2) |
|  | 365 | 400 µW cm^-2^ |  |  | (3) |
|  | 365 | 400 µW cm^-2^ |  |  | (4) |
|  |  |  | 302 | 20 kJ m^-2^ | (5) |
|  |  |  | 300 | 5 kJ m^-2^ | (6) |
|  |  |  | 300 | 8 kJ m^-2^ | (7) |
| Rat in vivo |  |  | 300 | 8 kJ m^-2^ | (8) |
|  |  |  | 280-315 | 3.18-6.36 kJ cm^-2^ | (9) |
|  |  |  | 300 | 90 kJ m^-2^ | (10) |
|  |  |  | 300 | 2.5-7.5 kJ m^-2^ | (11) |
|  |  |  | 300 | 16 kJ m^-2^ | (12) |
|  |  |  | 300 | 90 kJ m^-2^ | (13) |
|  |  |  | 302 | 5 kJ m^-2^ | (14) |
|  |  |  | 300 | 5 kJ m^-2^ | (15) |
|  |  |  | 300 | 8 kJ m^-2^ | (16) |
|  |  |  | 302 | 8 kJ m^-2^ | (17) |
|  |  |  | 300 | 5-8 kJ m^-2^ | (18) |
|  |  |  | 300 | 1 kJ m^-2^ | (19) |
|  |  |  | 300 | 5-20 kJ m^-2^ | (20) |
|  |  |  | 300 | 30 kJ m^-2^ | (21) |
| Rat ex vivo | 360 | 6 mW cm^-2^ |  |  | (22) |
|  |  |  | 300 | 100 µW cm^-2^ | (23) |
|  |  |  | 300 | 100 µW cm^-2^ | (24) |
|  |  |  | 300 | 100 µW cm^-2^ | (25) |
|  |  |  | 302 | 1.8 kJ m^-2^ | (26) |
| Guinea pig in vivo | 340-410 | 0.5 mW cm^-2^ |  |  | (27) |
|  | 353 | 1.3 mW cm^-2^ |  |  | (28) |
|  | 340-410 | 0.5 mW cm^-2^ |  |  | (29) |
|  |  |  | 300 | <84.9 kJ cm^-2^ | (30) |
|  | 305-410 | 4 mW cm^-2^ |  |  | (31) |
| Guinea pig ex vivo | 353 | 90 mW cm^-2^ |  |  | (28) |
| Rabbit in vivo | 365 | 0.589 J cm^-2^ |  |  | (32) |
|  | 365 | 5-10 J cm^-2^ |  |  | (33) |
|  |  |  | 295-315 | <6 J cm^-2^ | (34) |
|  |  |  | 315 | 3.12 J cm^-2^ | (35) |
|  | 365 | 100 mW cm^-2^ |  |  | (36) |
| Rabbit ex vivo | 365 | 2.5 mW cm^-2^ |  |  | (37) |
|  | 330-400 | 6-24 J cm^-2^ | 295-330 | 3 J cm^-2^ | (38) |
|  |  |  | 311 | 4 J cm^-2^ | (39) |
| Squirrel in vivo | 365 | 6 mW cm^-2^ |  |  | (40) |
|  | 365 | 1.3 mW cm^-2^ |  |  | (41) |
| Squirrel ex vivo | 365 | 5 mW cm^-2^ |  |  | (41) |
|  |  |  | 300 | 10 µW cm^-2^ | (42) |
|  | 365 | 1.53 mW cm^-2^ |  |  | (43) |
| Pig ex vivo | 350 | 222.6 J cm^-2^ |  |  | (44) |
|  | 365 | 86 J cm^-2^ | 280-290 | 0.12 J cm^-2^ | (45) |
| Cow ex vivo | 365 | 8.5 mW cm^-2^ |  |  | (46) |
|  | 365 | 4 J cm^-2^ |  | 0.06 J cm^-2^ | (47) |
|  | 365 | 33 J cm^-2^ |  |  | (48) |
|  | 365 | 4 J cm^-2^ |  |  | (49) |
|  |  |  | 300 | 0.5 J cm^-2^ | (50) |

1. Jose JG. Posterior cataract induction by UV-B radiation in albino mice. Experimental Eye Research. 1986;42:11-20.

2. Jose JG, Pitts DG. Wavelength dependency of cataracts in albino mice following chronic exposure. Experimental Eye Research. 1985;41:545-63.

3. Zigman S, Griess G, Yulo T, Schultz J. Ocular Protein Alterations by Near UV Light. Experimental Eye Research. 1973;15:255-64.

4. Zigman S, Yulo T, Schultz J. Cataract Induction in Mice Exposed to Near UV Light. Ophthalmic Research. 1974;6(2-4):259-70.

5. Zhang J, Yan H, Löfgren S, Tian X, Lou MF. Ultraviolet Radiation–Induced Cataract in Mice: The Effect of Age and the Potential Biochemical Mechanism. Investigative Opthalmology & Visual Science. 2012;53(11):7276.

6. Meyer LM, Söderberg P, Dong X, Wegener A. UVR-B induced cataract development in C57 mice. Experimental Eye Research. 2005;81.

7. Meyer LM, Löfgren S, Ho Y-S, Lou MF, Wegener A, Holz F, et al. Absence of glutaredoxin1 increases lens susceptibility to oxidative stress induced by UVR-B. Experimental Eye Research. 2009;89:833-9.

8. Galichanin K, Löfgren S, Bergmanson J, Söderberg PG. Evolution of damage in the lens after in vivo close to threshold exposure to UV-B radiation: Cytomorphological study of apoptosis. Experimental Eye Research. 2010;91:369-77.

9. Galichanin K, Löfgren S, Söderberg PG. Cataract after Repeated Daily in vivo Exposure to Ultraviolet Radiation. Health Physics. 2014;107(6):523-9.

10. Löfgren S, Söderberg PG. Lens Lactate Dehydrogenase Inactivation after UV-B Irradiation: An In Vivo Measure of UVR-B Penetration. Investigative Ophthalmology & Visual Science. 2001;42(8):1833-6.

11. Risa Ø, Sæther O, Löfgren S, Söderberg PG, Krane J, Midelfart A. Metabolic Changes in Rat Lens after In Vivo Exposure to Ultraviolet Irradiation: Measurements by High Resolution MAS1H NMR Spectroscopy. Investigative Opthalmology & Visual Science. 2004;45(6):1916.

12. Risa Ø, Sæther O, Kakar M, Mody VC, Löfgren S, Söderberg PG, et al. Time dependency of metabolic changes in rat lens after in vivo UVB irradiation analysed by HR-MAS 1H NMR spectroscopy. Experimental Eye Research. 2005;81(4):407-24.

13. Löfgren S, Söderberg PG. Rat lens glycolysis after in vivo exposure to narrow band UV or blue light radiation Journal of Photochemistry and Photobiology B: Biology. 1995;30:145-51.

14. Löfgren S, Michael R, Söderberg P. Impact of iris pigment and pupil size in ultraviolet radiation cataract in rat. Acta Ophthalmologica. 2010;90(1):44-8.

15. Michael R, Vrensen GFJM, van Marle J, Gan L, Söderberg PG. Apoptosis in the rat lens after in vivo threshold dose ultraviolet irradiation. Investigative Ophthalmology & Visual Science. 1998;39(13):2681-7.

16. Ayala M, Strid H, Jacobsson U, Söderberg PG. p53 Expression and Apoptosis in the Lens after Ultraviolet Radiation Exposure. Investigative Opthalmology & Visual Science. 2007;48(9):4187.

17. Dong X, Ayala M, Löfgren S, Söderberg PG. Ultraviolet Radiation–Induced Cataract: Age and Maximum Acceptable Dose. Investigative Opthalmology & Visual Science. 2003;44(3):1150.

18. Löfgren S, Michael R, Söderberg PG. Impact of Age and Sex in Ultraviolet Radiation Cataract in the Rat. Investigative Opthalmology & Visual Science. 2003;44(4):1629.

19. Talebizadeh N, Yu Z, Kronschläger M, Söderberg P. Modelling the Time Evolution of Active Caspase-3 Protein in the Rat Lens after In Vivo Exposure to Ultraviolet Radiation-B. PLoS ONE. 2014;9(9):e106926.

20. Michael R, Söderberg PG, Chen E. Long-term development of lens opacities after exposure to ultraviolet radiation at 300 nm. Ophthalmic Research. 1996;28:209-18.

21. Söderberg PG. Development of light dissemination in the rat lens after *in vivo* exposure to radiation in the 300 nm wavelength region. Ophthalmic Research. 1990;22:271-9.

22. Torriglia A, Zigman S. The effect of near-UV light on Na-K-ATPase of the rat lens. Current Eye Research. 1988;7(6):539-48.

23. Reddy B, Bhat S. Synergistic effect of UVB radiation and age on HMPS enzymes in rat lens homogenate Journal of Photochemistry and Photobiology B: Biology. 1998;43:56-60.

24. Reddy VN, Giblin FJ, Lin L-R, Chakrapani B. The Effect of Aqueous Humor Ascorbate on Ultraviolet B-Induced DNA Damage in Lens Epithelium. Investigative Ophthalmology & Visual Science. 1998;39(2):344-50.

25. Reddy B, Bhat S. Protection against UVB inactivation (in vitro) of rat lens enzymes by natural antioxidants. Molecular and Cellular Biochemistry. 1999;194:41-5.

26. Löfgren S. Lenses from Brown-Norway pigmented rats are more tolerant to in vitro ultraviolet irradiation than lenses from Fischer-344 albino rats. Acta Ophthalmologica. 2012;90(2):179-83.

27. Giblin FJ, Leverenz VR, Padgaonkar VA, Unakar NJ, Dang L, Lin L-R, et al. UVA Light In vivo Reaches the Nucleus of the Guinea Pig Lens and Produces Deleterious, Oxidative Effects. Experimental Eye Research. 2002;75(4):445-58.

28. Barron BC, Yu N-T, Kuck JFRJ. Raman Spectroscopic Evaluation of Aging and Long-wave UV Exposure in the Guinea Pig Lens: A Possible Model for Human Aging. Experimental Eye Research. 1988;46:249-58.

29. Simpanya MF, Ansari RR, Leverenz VR, Giblin FJ. Measurement of lens protein aggregation in vivo using dynamic light scattering in a guinea pig/UVA model for nuclear cataract. Photochemistry and Photobiology. 2008;84:1589-95.

30. Mody VC, Kakar M, Söderberg PG, Löfgren S. High lenticular tolerance to ultraviolet radiation-B by pigmented guinea pig; application of a safety limit strategy for UVR induced cataract. Acta Ophthalmologica. 2012;90:226-30.

31. Bergbauer KL, Kuck JFR, Su KC, Yu N-T. Use of a UV-Blocking contact lens in evaluation of UV-induced damage to the guinea pig lens. International Contact Lens Clinic. 1991;18(9-10):182-7.

32. Tessem M-B, Midelfart A, Čejková J, Bathen TF. Effect of UVA and UVB Irradiation on the Metabolic Profile of Rabbit Cornea and Lens Analysed by HR-MAS 1H NMR Spectroscopy. Ophthalmic Research. 2006;38(2):105-14.

33. Čejka Č, Pláteník J, Buchal R, Guryca V, Širc J, Vejražka M, et al. Effect of Two Different UVA Doses on the Rabbit Cornea and Lens. Photochemistry and Photobiology. 2009;85(3):794-800.

34. Pitts DG, Cullen AP, Hacker PD. Ocular effects of ultraviolet radiation from 295 to 365 nm. Investigative Ophthalmology & Visual Science. 1977;16(10):932-9.

35. Fris M, Čejková J, Midelfart A. The effect of single and repeated UVB radiation on rabbit lens. Graefe's Archive for Clinical and Experimental Ophthalmology. 2008;246(4):551-8.

36. Giblin FJ, Lin L-R, Simpanya MF, Leverenz VR, Fick CE. A Class I UV-blocking (senofilcon A) soft contact lens prevents UVA-induced yellow fluorescence and NADH loss in the rabbit lens nucleus in vivo. Experimental Eye Research. 2012;102:17-27.

37. Rafferty NS, Zigman S, McDaniel T, Scholz DL. Near-UV radiation disrupts filamentous actin in lens epithelial cells. Cell Motility and the Cytoskeleton. 1993;26(1):40-8.

38. Hightower K, McCready J. Comparative effect of UVA and UVB on cultured rabbit lens Photochemistry and Photobiology. 1993;58(6):827-30.

39. Hightower KR, McCready J. Physiological effects of UVB irradiation on cultured rabbit lens. Investigative Ophthalmology & Visual Science. 1992;33(5):1783-7.

40. Zigman S, Paxhia T, McDaniel T, Lou MF, Yu N-T. Effect of chronic near-ultraviolet radiation on the gray squirrel lens in vivo. Investigative Ophthalmology & Visual Science. 1991;32(6):1723-32.

41. Zigman S, Paxhia T, Waldron W. Effects of near-UV radiation on the protein of the grey squirrel lens. Current Eye Research. 1988;7(6):531-7.

42. Nagalaxmi V, Praveen KM, Sashidhar R, Turlapati NR. UV-B Exposure Increases the Activity of Indoleamine 2, 3-Dioxygenase (Ido) and Alters the Levels of Tryptophan Metabolites in Indian Ground Squirrel (Funambulus Palmarum) Lens. Journal of Diabetic Complications & Medicine. 2015;1(1):6.

43. Thomas DM, Papadopoulou O, Mahendroo PP, Zigman S. Phosphorous-31 NMR Study of the Effects of UV on Squirrel Lenses. Experimental Eye Research. 1993;57:59-65.

44. Oriowo OM, Cullen AP, Chou BR, Sivak JG. Action spectrum and recovery for in vitro UV-induced cataract using whole lenses. Investigative Ophthalmology & Visual Science. 2001;42(11):2596-602.

45. Oriowo OM, Cullen AP, Sivak JG. Impairment of Eye Lens Cell Physiology and Optics by Broadband Ultraviolet A-Ultraviolet B Radiation. Photochemistry and Photobiology. 2002;76(3):361-7.

46. Weinreb O, Adrianus M, van Boekel M, Dovrat A, Bloemendal H. Effect of UV-A Light on the Chaperone-like Properties of Young and Old Lens α-Crystallin. Investigative Ophthalmology & Visual Science. 2000;41(1):191-8.

47. Stuart DD, Cullen AP, Sivak JG, Doughty MJ. Optical effects of UV-A and UV-B radiation on the cultured bovine lens. Current Eye Research. 1994;13(5):371-6.

48. Dovrat A, Weinreb O. Effects of UV-A radiation on lens epithelial NaK-ATPase in organ culture. Investigative Ophthalmology & Visual Science. 1999;40:1616-20.

49. Dovrat A, Weinreb O. Recovery of Lens Optics and Epitehlial Enzymes After Ultraviolet A Radiation. Investigative Ophthalmology & Visual Science. 1995;36(12):2417-24.

50. Stuart DD, Sivak JG, Cullen AP, Weerheim JA, Monteith CA. UV-B radiation and the optical properties of cultured bovine lenses. Current Eye Research. 1991;10(2):177-84.
